# Prenatal Maternal Psychological Distress During the COVID-19 Pandemic and Newborn Brain Development

**DOI:** 10.1001/jamanetworkopen.2024.17924

**Published:** 2024-06-20

**Authors:** Susan Weiner, Yao Wu, Kushal Kapse, Tracy Vozar, Jenhao Jacob Cheng, Jonathan Murnick, Diedtra Henderson, Hironori Teramoto, Catherine Limperopoulos, Nickie Andescavage

**Affiliations:** 1 Developing Brain Institute, Children’s National Hospital, Washington, DC; 2 The Institute for Biomedical Sciences, George Washington University, Washington, DC; 3 Department of Diagnostic Imaging and Radiology, Children’s National Hospital, Washington, DC; 4 Department of Psychology, Children’s National Hospital, Washington, DC; 5 Department of Pediatrics, George Washington University, Washington, DC; 6 Department of Radiology, George Washington University, Washington, DC; 7 Department of Neonatology, Children’s National Hospital, Washington, DC

## Abstract

**Importance:**

Elevated maternal psychological distress during pregnancy is associated with altered fetal brain development. During the COVID-19 pandemic, prenatal maternal psychological distress more than doubled.

**Objective:**

To examine the association of the pandemic and rising maternal psychological distress with brain growth in newborns using quantitative 3-dimensional volumetric magnetic resonance imaging (MRI).

**Design, Setting, and Participants:**

This prospective cross-sectional study recruited mother-infant dyads at Children’s National Hospital, Washington, DC, during the COVID-19 pandemic (June 1, 2020, to June 30, 2022) into a longitudinal infant brain development study and compared them with an existing normative healthy cohort (recruited March 1, 2014, to December 31, 2019). Exclusion criteria included multiple gestation pregnancy, known or suspected congenital infection, documented chromosomal abnormalities, or any maternal contraindication to MRI, as well as prenatal COVID-19 exposure. Infants with structural brain abnormalities or a postnatal confirmation of a genetic syndrome were excluded.

**Exposure:**

Psychological distress during COVID-19 pandemic.

**Main Outcomes and Measures:**

Prenatal maternal mental health was evaluated using the Spielberger State-Trait Anxiety Inventory and the Perceived Stress Scale. Neonates underwent nonsedated brain MRI. An ordinary least squares linear regression model was used to measure the differences in regional brain volumes of neonates born before vs during the pandemic with and without exposure to elevated prenatal maternal psychological distress after adjustment for neonatal sex and gestational age at MRI and maternal age and educational level.

**Results:**

A total of 159 mother-infant dyads were included in the analysis: 103 before and 56 during the pandemic (median gestational age of infants, 39.6 [IQR, 38.4-40.4] weeks; median maternal age, 34.5 [IQR, 31.0-37.0] years). Eighty-three infants (52.2%) were female. Among the mothers, 130 (81.8%) had a college degree and 87 (54.7%) had a graduate degree. Forty-four mothers (27.7%) identified as Asian, Hispanic, or multiracial; 27 (17.0%), as Black; and 88 (55.3%), as White. Scores on anxiety and stress measures were significantly increased in the pandemic cohort. Infants of mothers with elevated maternal distress showed median reductions in white matter (−0.36 [95% CI, −0.61 to −0.11] cm^3^; *Q* < .001), right hippocampal (−0.35 [95% CI, −0.65 to −0.06] cm^3^; *Q* = .04), and left amygdala (−0.49 [95% CI, −0.84 to −0.13] cm^3^; *Q* = .03) volumes compared with infants of mothers with low distress levels. After adjusting for the cohort effect of the pandemic, elevated trait anxiety remained significantly associated with decreased left amygdalar volumes (−0.71 [95% CI, −1.12 to −0.29]; *Q* < .001).

**Conclusions and Relevance:**

In this cross-sectional study of maternal-infant dyads prior to and during the COVID-19 pandemic, regional neonatal brain volumes were associated with elevated maternal psychological distress.

## Key Points

**Question:**

Is increased maternal psychological distress during the COVID-19 pandemic associated with neonatal brain development?

**Findings:**

In this cross-sectional study of 159 mother-infant dyads, left amygdalar volumes were smaller in neonates born to mothers with high psychological distress during the pandemic.

**Meaning:**

These findings suggest that increased maternal mental health symptoms during the COVID-19 pandemic are associated with subsequent changes in regional brain growth in newborn offspring.

## Teaser

This cross-sectional study examines the association of prenatal maternal mental health during the COVID-19 pandemic, in the absence of direct viral exposure or infection, with neonatal brain development in a cohort of mother-infant dyads.

## Introduction

Intrauterine stressors have been shown to influence fetal brain development and affect how the child, once born, grows and develops.^1,2,3^ Maternal psychological distress during pregnancy, including stress, anxiety, and depression, is recognized as one such stressor on early brain development,^4,5,6,7,8,9,10,11,12^ as well as psychosocial adversity such as trauma, bereavement, or poverty.^13,14^ Available evidence shows that maternal anxiety during pregnancy is associated with altered brain growth and microstructure development in the amygdala and white matter in the offspring,^8,9,10,12,15,16^ as well as lower cognitive scores and increased negative temperament from infancy to 2 years of age.^17,18,19^ Since 2020, several groups^4,5,7,9^ have reported that alterations in offspring brain growth and metabolism in the setting of maternal psychological distress detected in utero are associated with offspring behavior at 18 months.^6^

During the COVID-19 pandemic, the world experienced an alarming increase in the incidence of psychological distress,^8,9,10^ including among pregnant women.^20,21,22,23,24,25,26,27,28^ Lu et al^4^ previously demonstrated that fetal brain development during the COVID-19 pandemic was associated with decreased cerebral white matter, hippocampal, and cerebellar volumes, even in the absence of maternal COVID-19 exposure. However, the potential enduring effects of these prenatal alterations in fetal brain development in the offspring remain unclear. The magnitude and extent of maternal psychological distress experienced during the COVID-19 pandemic raise the possibility of a secondary epidemic of neurobehavioral concerns in the next generation of children born during this period. The objective of our study was to determine the association of prenatal maternal mental health during the COVID-19 pandemic, in the absence of direct viral exposures or infection, with neonatal brain development, using the same cohort as in the previous fetal study.^4^ We hypothesized that elevated maternal psychological distress during pregnancy would result in smaller regional and tissue-specific brain volumes measured in the neonatal period.

## Methods

### Study Participants

Mother-infant dyads were prospectively recruited for longitudinal brain magnetic resonance imaging (MRI) studies, including a fetal and a neonatal scan. For this cross-sectional study, imaging performed during the neonatal period is reported.^4^ Acquisition protocols for MRI and maternal assessments were identical across cohorts. Exclusion criteria included multiple gestation pregnancy, known or suspected congenital infection, documented chromosomal abnormalities, or maternal contraindication to MRI. To disentangle potential effects of COVID-19 exposures, mothers with documented COVID-19 infections were excluded in this report; COVID-19 exposures were determined through serial self-reported surveys and medical records. Additionally, mothers reporting the use of medications or substances other than prenatal vitamins or supplements were excluded (eg, prescribed medications, tobacco, marijuana, or alcohol). Enrolled infants found to have structural brain abnormalities on fetal or neonatal MRIs or a postnatal confirmation of a genetic syndrome were subsequently excluded from the study. Demographic information, including maternal age, educational level, and race and ethnicity were collected via self-reported assessments by the mother. Given the relatively low numbers of participants who reported racial background other than Black or White, additional racial and ethnic categories, including multiracial and Hispanic ethnicity, were reported as *other*. All study procedures for the prepandemic and pandemic cohorts were reviewed and approved by the institutional review board at Children’s National Hospital, Washington, DC. Written informed consent was obtained from all participants. This study followed the Strengthening the Reporting of Observational Studies in Epidemiology (STROBE) reporting guidelines for cross-sectional studies.^29^

### Maternal Distress

Each pregnant woman completed previously validated maternal distress measures during an onsite fetal visit, including the Spielberger State Anxiety Inventory (SSAI; score range, 20-80),^30^ the Spielberger Trait Anxiety Inventory (STAI; score range, 20-80),^30^ and the Perceived Stress Scale (PSS; score range, 0-40).^31^ Scores on the SSAI greater than 40, on the STAI greater than 40, and on the PSS greater than 15 were considered elevated.^31,32,33^ Mothers who scored above these thresholds on 1 or more assessments were considered psychologically distressed.

### MRI Data Acquisition

Neonatal MRI studies were performed on a 3T MR scanner (Discovery MR750; GE Healthcare) and an 8-channel high-resolution brain array receive-only head coil (T2-weighted, 3-dimensional [3D] CUBE sequence; repetition time, 2500 milliseconds; echo time, 64.7 milliseconds; flip angle, 90°; 0.625 × 0.625 × 1 mm^3^). Acquisition time was 3 to 4 minutes. Infants were scanned during natural sleep after being fed, swaddled in warm blankets, and immobilized with a vacuum pillow to prevent the need for sedation.

### Image Processing

A quality assessment of each scan for signs of movement of the participant was conducted before analysis, and 3D brain images with severe motion artifacts that affected the ability to distinguish brain tissues such as the cortical gray mater, white matter, brainstem, and cerebellum were excluded from analysis. Postacquisition processing of volumetric brain measurements was performed using the DrawEM (Developing Brain Region Annotation With Expectation-Maximization [MIRTK]) software tool and 3D U-Net–based methods that have been validated in newborns.^34^ Regions of interest were determined based on prior reports and included the cerebral white matter, left and right hippocampus, left and right amygdala, and cerebellum,^4,5,7,34^ where the cerebral white matter and cerebellum were automatically segmented by DrawEM images^35^ and bilateral hippocampus and amygdala were automatically segmented by 3D U-Net images,^36,37^ and all regions were manually corrected using open-source ITK-SNAP software in the coronal, sagittal, and axial fields. A representative image of this 3D reconstruction is shown in eFigure 1 in Supplement 1.

### Statistical Analysis

All analyses were performed with R statistical software, version 4.3.0 (R Program for Statistical Computing). We first applied the descriptive analysis to demographic characteristics and maternal psychological distress measures to report their distributions by median and IQR for continuous variables, as they all failed to pass the Shapiro-Wilk normality tests, and by frequency and percentage for categorical variables. The cohort differences for these demographic characteristics and distress measures were assessed using Wilcoxon rank sum tests for continuous data and χ^2^ tests for categorical data. Multivariable linear regression models based on ordinary least squares were used to evaluate the association between the neonatal brain development and pandemic cohort type, psychological distress measure(s), and neonatal sex. For each specific covariate, separate regression models were conducted for different brain volume outcomes (measured from 6 regions) with the following covariates included for adjustment: gestational age at MRI scan, neonatal sex, maternal age, and maternal educational level. Neonatal sex was not regarded as an adjusting covariate if it was already the primary covariate, and the associations (or mean differences) were estimated by the standardized coefficients. Statistical significance was considered as *P* < .05 for a 2-sided test. The Benjamini-Hochberg method was used to adjust for multiple testing where the correction was applied to 6 model-based tests for each covariate, since multiple outcomes by region were compared. To differentiate from the raw *P* values, the adjusted *P* values were labeled as *Q* values.

## Results

### Study Population

This study prospectively enrolled 223 mother-infant dyads. One hundred three dyads were recruited from a prepandemic normative healthy cohort (March 1, 2014, to December 31, 2019), and 120 were recruited during the COVID-19 pandemic (June 1, 2020, to June 30, 2022). Of these, 44 were excluded due to confirmed COVID-19 infection (n = 29) or potential exposure (n = 15) (eFigure 2 in Supplement 1). An additional 20 dyads were excluded for either not having an MRI scan (n = 15) or for having excessive motion during the scan (n = 5) (eFigure 2 in Supplement 1). Table 1 summarizes participant demographic characteristics. Median gestational age of the infants was 39.6 (IQR, 38.4-40.4) weeks; 83 (52.2%) were female and 76 (47.8%) were male. Median maternal age was 34.5 (IQR, 31.0-37.0) years; 44 (27.7%) identified as Asian, Hispanic, or multiracial; 27 (17.0%), Black; and 88 (55.3%), White. One hundred thirty mothers (81.8%) had a college and/or graduate degree and 112 (70.4%) were employed as business professionals. There were no significant differences between the 2 cohorts in maternal age, educational level, or employment or in neonate sex, gestational age, head circumference, or weight at birth (Table 1). The pandemic cohort underwent a postnatal MRI at an older gestational age than the prepandemic cohort (44.9 [IQR, 43.3-46.9] vs 41.9 [IQR, 40.4-43.0] weeks); thus, gestational age at MRI was considered and adjusted for in all analyses. We successfully acquired 159 MRI scans (103 from the prepandemic cohort and 56 from the pandemic cohort).

**Table 1.** Demographic Characteristics

Characteristic	Mother-infant dyads by cohort^a^			*P* value	No. with data collected
	All (N = 159)	Prepandemic (n = 103)	Pandemic (n = 56)		
Neonatal sex					
Female	83 (52.2)	53 (51.5)	30 (53.6)	.93	159
Male	76 (47.8)	50 (48.5)	26 (46.4)		
GA at scan, median (IQR), wk	42.6 (40.9-44.5)	41.9 (40.4-43.0)	44.9 (43.3-46.9)	<.001	151
GA at birth, median (IQR), wk	39.6 (38.4-40.4)	39.6 (38.3-40.3)	39.7 (38.7-40.4)	.31	159
Birth weight, median (IQR), kg	3.35 (3.00-3.65)	3.34 (3.00-3.65)	3.35 (3.06-3.66)	.73	159
Head circumference, median (IQR), cm	34.0 (33.0-35.5)	34.0 (33.0-35.5)	34.0 (33.0-35.5)	.89	133
Maternal age, median (IQR), y	34.5 (31.0-37.0)	34.5 (31.0-38.0)	34.0 (30.7-36.0)	.68	156
Maternal race and ethnicity					
Black	27 (17.0)	17 (16.5)	10 (17.9)		
White	88 (55.3)	61 (59.2)	27 (48.2)		
Other^b^	44 (27.7)	25 (24.3)	19 (33.9)		
Maternal educational level					
High school	29 (18.2)	19 (18.4)	10 (17.9)	.69	159
College	43 (27.0)	30 (29.1)	13 (23.2)		
Graduate school	87 (54.7)	54 (52.4)	33 (58.9)		
Maternal employment					
Major professional	112 (70.4)	77 (74.8)	35 (62.5)	.15	159
Minor and labor	47 (29.6)	26 (25.2)	21 (37.5)		

Abbreviation: GA, gestational age.

^a^ Unless otherwise specified, data are expressed as No. (%) of participants.

^b^ Includes participants who reported being Asian, Hispanic, or multiracial individuals.

### Maternal Anxiety, Stress, and Depression

 Scores on the SSAI, STAI, and PSS were significantly higher in the pandemic cohort than in the prepandemic cohort (Table 2). Among women in the prepandemic cohort, 20 of 95 (21.1%) scored above the threshold for anxiety (state, 12 of 94 [12.8%]; trait, 13 of 94 [13.8%]), and 23 of 93 (24.7%) scored positive for stress; overall, 27 of 95 mothers (28.4%) in the prepandemic cohort scored above the threshold on the pooled metric of psychological distress (a score above the threshold on ≥1 assessment). In the pandemic cohort, 34 of 55 mothers (61.8%) scored above the threshold for anxiety (state: 31 of 55 [56.4%]; trait: 25 of 55 [45.5%]), and 38 of 55 (69.1%) scored above the threshold for stress; overall, 40 of 55 mothers (72.7%) scored above the threshold for psychological distress. The proportion of women who scored above the threshold for each metric was significantly greater in the pandemic cohort than in the prepandemic cohort (*P* < .001) (Table 2).

**Table 2.** Maternal Psychological Distress Measures^a^

Measure^b^	Cohort^c^		
	All	Prepandemic	Pandemic
Median score (95% CI)			
SSAI	31.0 (24.0-40.0)	29.0 (23.0-34.8)	39.0 (31.2-48.8)
STAI	32.0 (26.0-40.0)	30.0 (25.0-36.0)	37.0 (30.0-44.5)
PSS	13.0 (9.00-17.0)	11.0 (7.00-15.0)	16.0 (12.0-21.0)
State anxiety			
Below threshold (<40)	106 (71.1)	82 (87.2)	24 (43.6)
Above threshold (≥40)	43 (28.9)	12 (12.8)	31 (56.4)
Trait anxiety			
Below threshold (<40)	111 (74.5)	81 (86.2)	30 (54.5)
Above threshold (≥40)	38 (25.5)	13 (13.8)	25 (45.5)
Anxiety			
Below threshold (<40)	96 (64.0)	75 (78.9)	21 (38.2)
Above threshold (≥40)	54 (36.0)	20 (21.1)	34 (61.8)
Stress			
Below threshold (<15)	87 (58.8)	70 (75.3)	17 (30.9)
Above threshold (≥15)	61 (41.2)	23 (24.7)	38 (69.1)
Maternal Distress Index			
Below threshold	83 (55.3)	68 (71.6)	15 (27.3)
Above threshold	67 (44.7)	27 (28.4)	40 (72.7)

Abbreviations: PSS, Perceived Stress Scale; SSAI, Spielberger State Anxiety Inventory; STAI, Spielberger Trait Anxiety Inventory.

^a^ For all comparisons between the prepandemic and pandemic groups for the SSAI, STAI, and PSS measures, *P* < .001. For all comparisons between those with psychological distress measure scores below the threshold vs those with scores above the threshold, *P* < .001.

^b^ Mothers who scored above the threshold were considered psychologically distressed.

^c^ Unless otherwise indicated, data are expressed as No. (%) of total participants with data available.

### Association Between Sex and Regional Brain Volumes

Across the 2 study cohorts, cerebral white matter volume was significantly smaller in female compared with male neonates (0.66 [95% CI, 0.42-0.91] cm^3^; *Q* < .001) (eFigure 3 in Supplement 1). There was no association with sex detected in bilateral hippocampal or amygdalar volumes, nor in the cerebellum. When each cohort was examined, male infants had significantly larger white matter volumes (0.55 [95% CI, 0.27-0.84] cm^3^; *Q* < .001) and right hippocampal volumes (0.46 [95% CI, 0.11-0.81] cm^3^; *Q* = .03) compared with female infants in the prepandemic cohort (eTable in Supplement 1). In the pandemic cohort, male infants showed larger white matter volumes than female infants (0.87 [95% CI, 0.42-1.31] cm^3^; *Q* < .001) (eTable in Supplement 1).

### Association Between Pandemic and Regional Brain Volume

**Figure 1.**
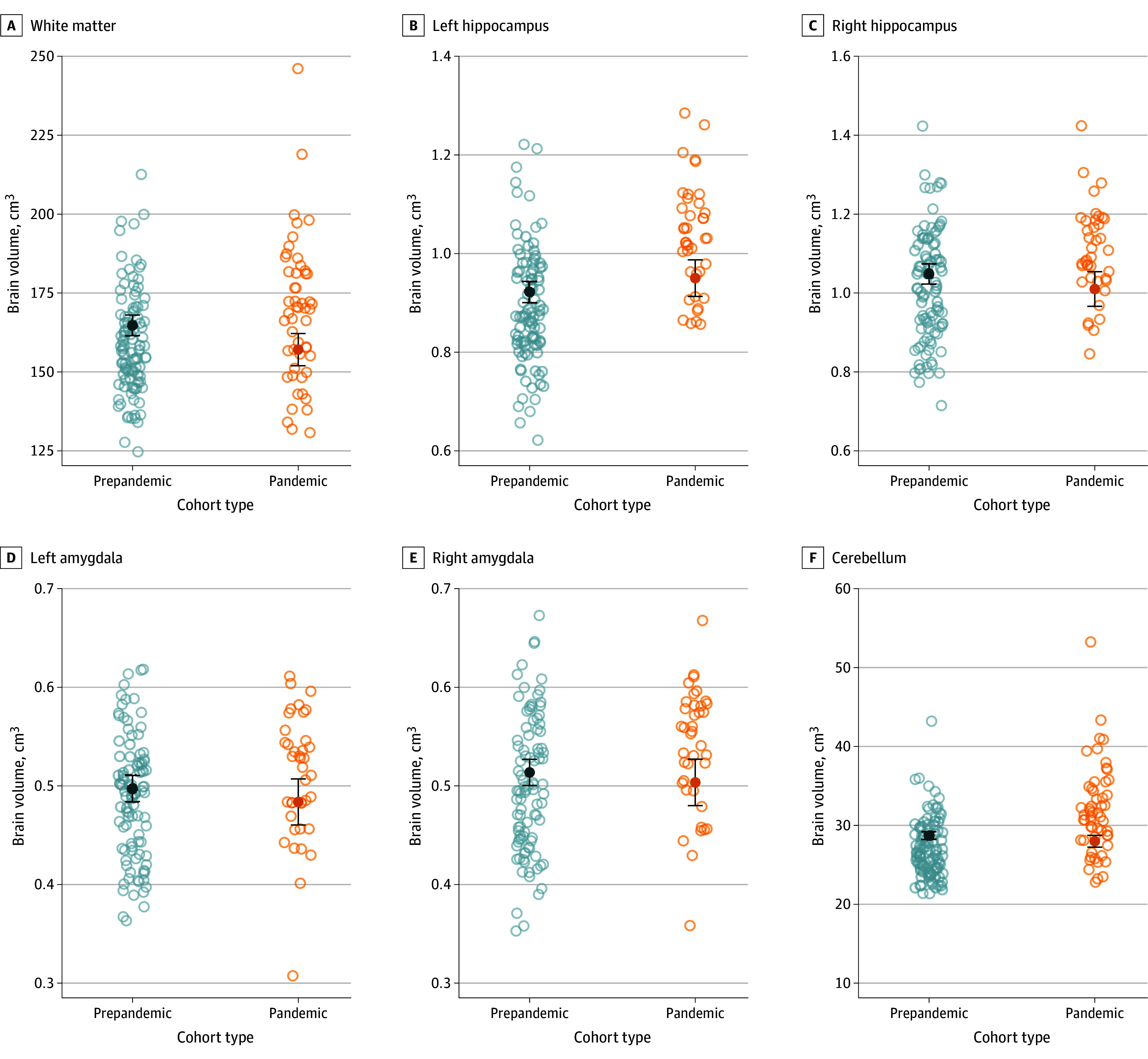
Comparisons of Neonatal Brain Volumes by Cohort Data represent regional brain volumes as a function of gestational age at magnetic resonance imaging scan, neonatal sex, maternal age, and maternal educational level. Open circles indicate individual measurements; solid circles, adjusted means; and error bars, 95% CIs.

Neonates born during the pandemic had significantly reduced white matter volumes compared with those born before the pandemic after adjustment for gestational age at MRI, neonatal sex, and maternal age irrespective of maternal mental health status, although this was attenuated following multiple comparison corrections (−0.41 [95% CI, −0.72 to −0.10] cm^3^; *P* = .01; *Q* = .06) (Figure 1). Hippocampal, amygdalar, and cerebellar volumes were not significantly different between prepandemic and pandemic cohorts.

### Association Between Maternal Distress and Regional Brain Volume

**Figure 2.**
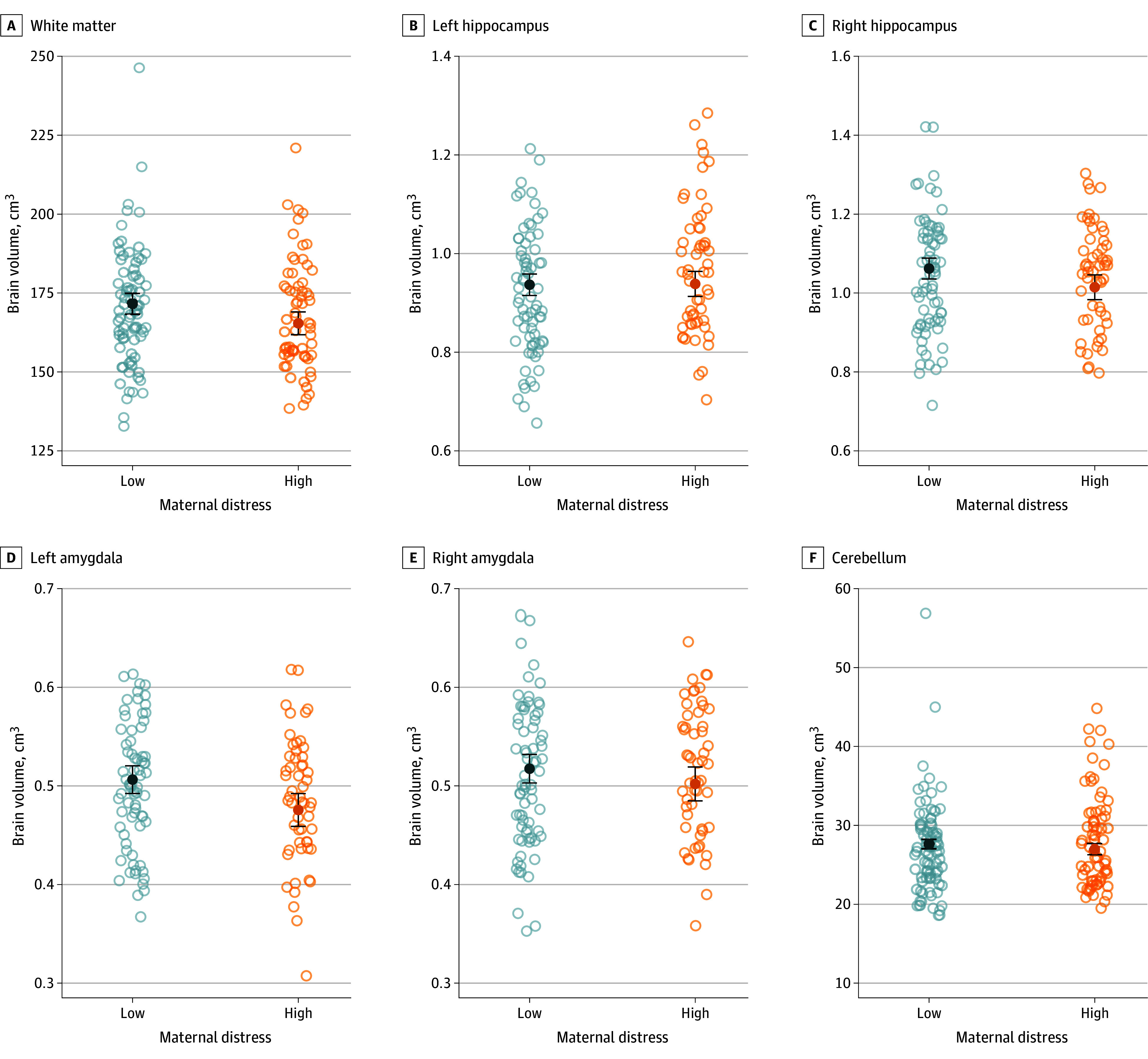
Comparisons of Neonatal Brain Volumes by Maternal Distress A pooled metric of maternal distress was used, with high level being defined as an elevated score on at least 1 of the maternal mental health assessments. Data represent regional brain volumes as a function of gestational age at magnetic resonance imaging scan, neonatal sex, maternal age, and maternal educational level. Open circles indicate individual measurements; solid circles, adjusted means; and error bars, 95% CIs.

Across both prepandemic and pandemic cohorts, infants of mothers with elevated psychological distress (using a pooled metric of elevated state-trait anxiety and/or stress scores) showed significantly smaller white matter (−0.36 [95% CI, −0.61 to −0.11] cm^3^; *Q* < .001), right hippocampal (−0.35 [95% CI, −0.65 to −0.06] cm^3^; *Q* = .04), and left amygdalar (−0.49 [95% CI, −0.84 to −0.13] cm^3^; *Q* = .03) volumes (Figure 2). There were no significant differences in left hippocampal, right amygdalar, or cerebellar volumes between infants of mothers with low or high maternal psychological distress levels.

### Cumulative Associations of Pandemic and Maternal Distress With Regional Brain Volume

After adjusting for pandemic effects, elevated maternal psychological distress was inversely associated with cerebral white matter (−4.94 [95% CI, −9.76 to −0.12] cm^3^; *P* = .04) and left amygdalar (−0.03 [95% CI, −0.05 to −0.01] cm^3^; *P* = .01) volumes in offspring (Table 3). More specifically, elevated maternal state anxiety was associated with decreased left amygdalar volume (−0.03 [95% CI, −0.06 to −0.01] cm^3^; *P* = .02), and elevated maternal trait anxiety was associated with decreased left amygdalar volume (−0.04 [95% CI, −0.07 to −0.02] cm^3^; *P* < .001) (Table 3). Elevated maternal stress was associated with smaller left amygdalar volumes (−0.03 [95% CI, −0.06 to −0.01] cm^3^; *P* = .01) (Table 3). After adjusting for multiple comparisons, left amygdalar volumes remained significantly lower for women with elevated trait anxiety (−0.71 [95% CI, −1.12 to −0.29] cm^3^; *Q* < .001) (Table 3).

**Table 3.** Association Between Maternal Psychological Distress With Regional Brain Volumes Adjusted for Pandemic Status

Region	Standardized mean difference, cm^3^ (95% CI)^a^	*P* value	*Q* value
Pooled maternal distress			
White matter	−0.27 (−0.54 to −0.01)	.04	.12
Left hippocampus	−0.03 (−0.30 to 0.24)	.84	.84
Right hippocampus	−0.30 (−0.61 to 0.01)	.06	.12
Left amygdala	−0.48 (−0.85 to −0.10)	.01	.06
Right amygdala	−0.21 (−0.57 to 0.14)	.24	.36
Cerebellum	−0.07 (−0.25 to 0.10)	.41	.49
State anxiety			
White matter	−0.22 (−0.53 to 0.08)	.15	.22
Left hippocampus	−0.08 (−0.40 to 0.24)	.63	.74
Right hippocampus	−0.30 (−0.67 to 0.07)	.11	.22
Left amygdala	−0.55 (−0.99 to −0.10)	.02	.12
Right amygdala	−0.42 (−0.84 to 0.00)	.05	.15
Cerebellum	−0.03 (−0.23 to 0.16)	.74	.74
Trait anxiety			
White matter	−0.25 (−0.54 to 0.05)	.10	.20
Left hippocampus	−0.20 (−0.50 to 0.10)	.19	.26
Right hippocampus	−0.22 (−0.58 to 0.14)	.22	.26
Left amygdala	−0.71 (−1.12 to −0.29)	<.001	<.001
Right amygdala	−0.41 (−0.80 to −0.01)	.04	.12
Cerebellum	−0.01 (−0.20 to 0.18)	.92	.92
Stress			
White matter	−0.17 (−0.45 to 0.11)	.22	.36
Left hippocampus	−0.08 (−0.37 to 0.20)	.55	.66
Right hippocampus	−0.28 (−0.61 to 0.05)	.09	.27
Left amygdala	−0.55 (−0.95 to −0.16)	.01	.06
Right amygdala	−0.22 (−0.59 to 0.15)	.24	.36
Cerebellum	−0.01 (−0.18 to 0.17)	.96	.96

^a^ Indicates difference between prepandemic and pandemic cohorts.

## Discussion

In this cross-sectional study, we report that neonates born during the COVID-19 pandemic demonstrated smaller cerebral white matter volumes compared with neonates in the prepandemic cohort. We also report regional decreases in cerebral white matter, right hippocampal, and left amygdalar volumes in offspring born during the pandemic to mothers with elevated prenatal maternal psychological distress. The COVID-19 pandemic negatively affected mental health globally, including pregnant women.^21,23^ Prior to the pandemic, maternal stress and anxiety during pregnancy were associated with decreased left hippocampal volumes in the fetus, with lasting effects on offspring behavior and development.^5,6,14^ Similarly, as previously reported in this cohort,^4^ the increase in maternal mental distress during the COVID-19 pandemic selectively stunted fetal cerebral white matter, hippocampal, and cerebellar growth. In the present study, we found persistent decrements in newborn cerebral white matter and right hippocampal volumes, especially in the pandemic cohort with high levels of stress and anxiety. These data underscore the putative role of heightened maternal stress on vulnerable brain regions during this critical period of prenatal brain development. We also report lower volumes of the left amygdala in neonates of mothers with elevated stress and anxiety during pregnancy that has not been observed prenatally. This finding may have been previously unseen given the smaller size and limited resolution of the amygdala on fetal imaging, which prevents accurate measurement and reporting. Conversely, we did not find differences in neonatal cerebellar growth, which has been reported in fetal scans,^4^ suggesting those differences were transient, subsequently accelerated, or subject to catch-up growth in the later part of gestation, resulting in no differences in cerebellar volumetric growth in the early postnatal period.

Our findings regarding white matter are noteworthy, given that alterations in white matter connectivity have been associated with anxiety disorders in the adult brain.^38,39,40,41^ Due to the early formation of white matter in the fetal brain, specifically of the emergence and formation of limbic tracts to the cingulum and the fornix,^42^ these white matter tracts may be particularly vulnerable to effects of prenatal maternal mental health.^13,15,43,44^ The fetal cingulum in particular has been shown to be affected by high levels of prenatal glucocorticoid exposure, such as in the case of prenatal stress. This period, specifically within the second trimester, is additionally the stage when early oligodendrocyte progenitor cells are developing and the process of myelination is beginning.^44,45^ Disruptions in this process decrease the volume of the white matter due to the lack of myelination, but also weaken potential connections. White matter remains susceptible to changes in the mental state of the mother throughout the perinatal period, which can explain why we see significant effects of stress and anxiety on fetal and neonatal scans.^4,6,10,13,15^

Our data show a selective vulnerability of the left amygdala in the newborn infant exposed to elevated maternal stress and anxiety during pregnancy. The amygdala is central to fear emotion processing in the brain and heavily implicated^46,47,48^ in anxiety, particularly in threat processing and anxiety, which have been linked to the left amygdala and its projections to the prefrontal cortex.^49^ We also report a significant decrease in the right amygdalar volumes in newborn offspring when exposed to elevated maternal trait anxiety, which reflects more stable expression of the personality trait of anxiety, as opposed to the more transient spikes of state anxiety.^30^ We posit differential susceptibility between chronic and acute exposures, with the right amygdala showing an association with chronic conditions or exposures. Published work demonstrating similar reductions in right amygdalar volume in the setting of other chronic maternal conditions, such as prenatal maternal depression, support this observation.^50^

Last, we report a differential effect between prenatal maternal state and trait anxiety and stress on neonatal brain development. Maternal mental health studies often evaluate the independent effects of stress, anxiety, depression, and trauma,^3,9,10,11,14,16,17,32,50,51^ despite the significant overlap in symptoms and high rates of mental health comorbidities. Despite these similarities, underlying biological mechanisms of stress and anxiety are likely mediated through distinct pathways^40,47,52,53,54^ and thus may exert unique effects on the offspring brain.^9,11,13,18,35,51,55,56,57,58^ Stress is thought to be predominately mediated by cortisol via the hypothalamic-pituitary-adrenal axis, whereas anxiety appears to be primarily mediated through dopamine circuits and receptors in the amygdala.^46,58,59,60,61^ Additionally, differences in duration of exposure may also influence early brain development, as seen in trait anxiety, which suggests chronic exposure compared with state anxiety and shorter-term exposures. The developmental window (and timing) of exposure likely results in distinct effects on offspring brain structure. The mechanisms driving the differences we report in regional brain development are undoubtedly complex, and the long-term implications require further study.

It is important to note that while psychological distress increased significantly during the pandemic, there remained effects of the pandemic on offspring brain development, independent of maternal mental health. Additional measures of adversity, particularly trauma or bereavement, as well as shifts in societal norms and increased social isolation,^62,63^ were unmeasured in this study. Similarly, the contribution of additional factors related to job insecurity and worries over family well-being, along with high levels of general unrest during this period, need to be considered. However, as the current and previous work suggest, there may be cumulative effects of both the pandemic and prenatal maternal mental distress that collectively affect offspring neurodevelopment. As none of the study participants in our pandemic cohort reported a diagnosis of COVID-19 during pregnancy, these differences are unlikely to be due to effects of viral exposure or transmission.

### Limitations

It is important to note the limitations of our study. First, the advent of the COVID-19 pandemic may have resulted in lifestyle changes that influenced both maternal mental health and infant development. Also, all mental health data were reported by the mothers, leading to potential self-reporting bias. Despite the rigorous screening of participants in this study to identify COVID-19 exposures, it is possible that they had unknown exposure or subclinical infection. This study included the greater Washington, DC, region and was open to pregnant women of all races, ethnicities, and resources. However, this study reported on a group of women predominantly of White race with high levels of education and employment. The experience of the participants in this study may not necessarily be representative of other communities or regions. Further, additional measures of psychosocial adversity have been shown to be associated with similar changes in brain development.^13,14,46,47^ These measures were beyond the scope of this work, but certainly warrant additional study. Last, there was a significant difference in gestational age at MRI; while gestational age at MRI was accounted for in our statistical models, these adjustments may not fully reflect the dynamic, and often nonlinear, nature of brain development during the neonatal developmental window.

## Conclusions

The findings of this cross-sectional study show an association between maternal psychological distress during the COVID-19 pandemic and offspring neonatal brain development. These findings suggest that increases in maternal mental health symptoms during the COVID-19 pandemic are associated with subsequent lower volumetric brain growth in newborn offspring. The extent to which these neonatal brain findings serve as early indices of risk for later social and emotional development are unknown. Further studies into the long-term impact on offspring development are needed and currently under way.

## Notes

Supplement 1. **eFigure 1.** Example of Volumetric Reconstruction **eFigure 2.** Excluded Participants **eFigure 3.** Sex Effect on Neonatal Brain Volumes **eTable.** Association Between Regional Brain Volumes and Sex by Cohort

Supplement 2.
Data Sharing Statement
