# Supplementary material for: Prenatal Maternal Psychological Distress During the COVID-19 Pandemic and Newborn Brain Development
Source: JAMA Netw Open. 2024 Jun 20;7(6):e2417924. doi: 10.1001/jamanetworkopen.2024.17924 (PMC11190810; doi:10.1001/jamanetworkopen.2024.17924)
Supplement: Supplement 1. — eFigure 1. Example of Volumetric Reconstruction eFigure 2. Excluded Participants eFigure 3. Sex Effect on Neonatal Brain Volumes eTable. Association Between Regional Brain Volumes and Sex by Cohort [file jamanetwopen-e2417924-s001.pdf]

Supplementary Online Content

Weiner S, Wu Y, Kapse K, et al. Prenatal maternal psychological distress during the COVID-19 pandemic and newborn brain development. *JAMA Netw Open*. 2024;7(6):e2417924. doi:10.1001/jamanetworkopen.2024.17924

eFigure 1. Example of Volumetric Reconstruction

eFigure 2. Excluded Participants

eFigure 3. Sex Effect on Neonatal Brain Volumes

eTable. Association Between Regional Brain Volumes and Sex by Cohort

This supplementary material has been provided by the authors to give readers additional information about their work.

© 2024 Weiner S et al. *JAMA Network Open*.

eFigure 1. Example of Volumetric Reconstruction

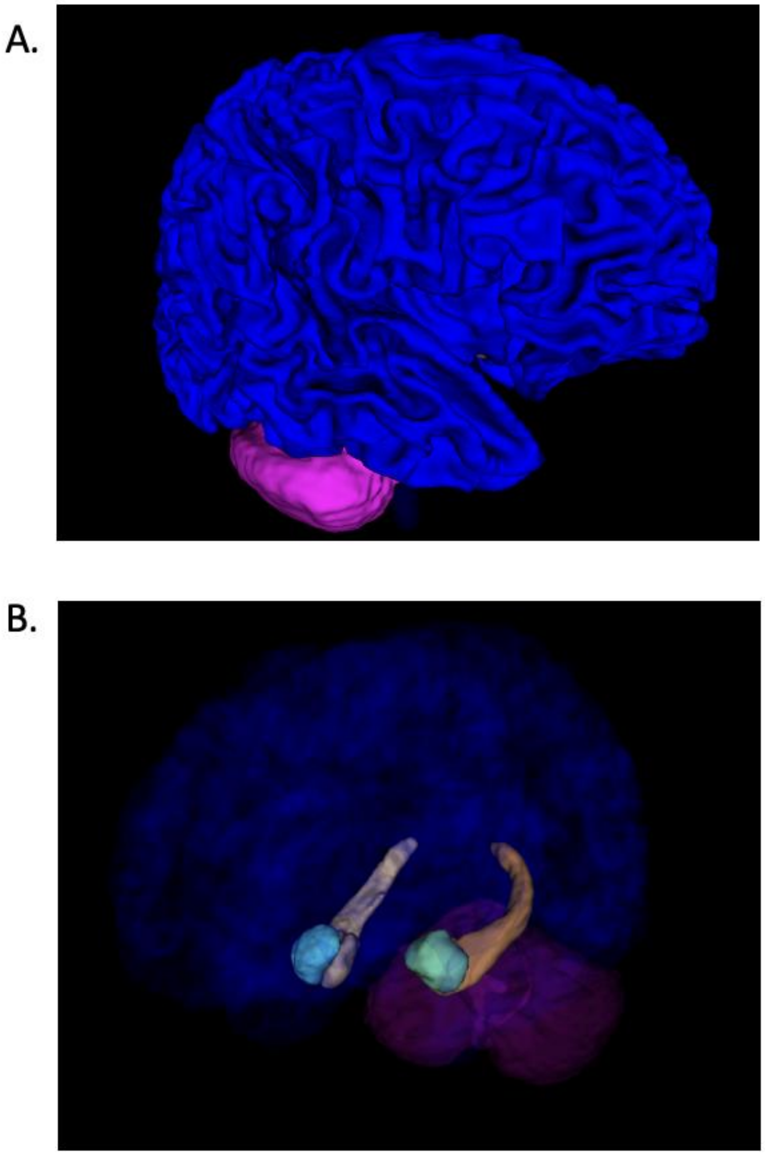

A representative image of brain reconstruction after segmentation using ITK-SNAP. A. Cerebellum is shown in pink. B. Left hippocampus (light beige), left amygdala (light blue), right hippocampus (brown) and right amygdala (light green).

© 2024 Weiner S et al. *JAMA Network Open*.

eFigure 2. Excluded Participants

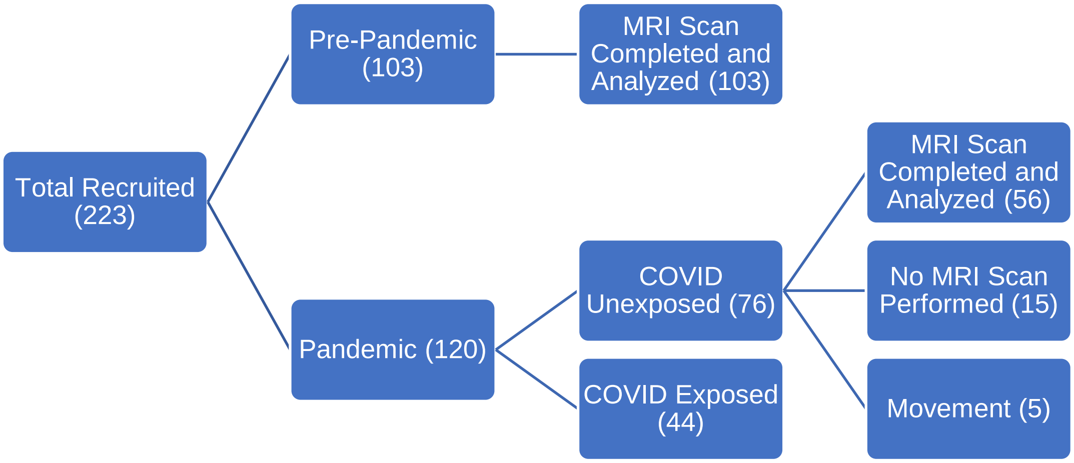

159 mother-baby dyads were prospectively enrolled in the study 103 from the prepandemic and 120 during the pandemic. 44 mothers during the pandemic were excluded for either confirmed or potential COVID infection. A further 15 more were removed for not completing a neonatal MRI scan. 5 additional subjects were excluded from study due to movement on the MRI scan, resulting in 56 scans being analyzed for the purposes of this study from the pandemic cohort.

© 2024 Weiner S et al. *JAMA Network Open*.

eFigure 3. Sex Effect on Neonatal Brain Volumes

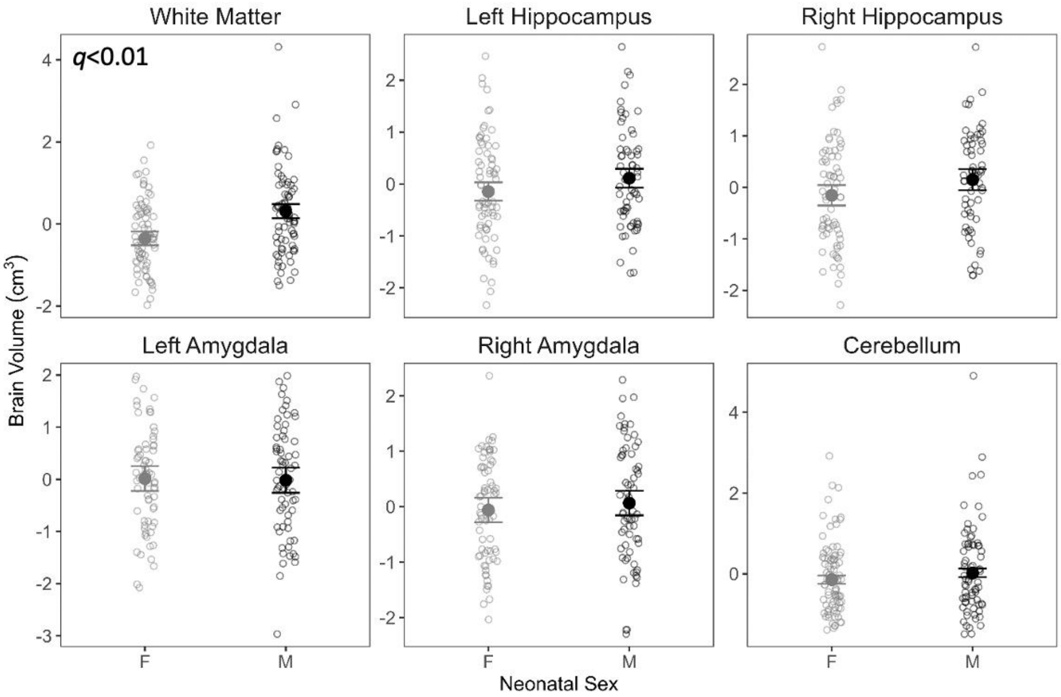

Sex effect on neonatal brain volumes. A. White Matter B. Left Hippocampus C. Right Hippocampus D. Left Amygdala E. Right Amygdala F. Cerebellum. Data shown as regional brain volumes as a function of gestational age. $q < 0.05$

© 2024 Weiner S et al. *JAMA Network Open*.

eTable. Association Between Regional Brain Volumes and Sex by Cohort

Cohort	Region	Mean diff (M vs F)	p	q
Prepandemic	White Matter	0.55 (0.27, 0.84)	0.00	0.00
Prepandemic	Left Hippocampus	0.33 (0.01, 0.65)	0.04	0.06
Prepandemic	Right Hippocampus	0.46 (0.11, 0.81)	0.01	0.03
Prepandemic	Left Amygdala	-0.03 (-0.40, 0.35)	0.89	0.89
Prepandemic	Right Amygdala	0.08 (-0.28, 0.44)	0.66	0.79
Prepandemic	Cerebellum	0.19 (0.00, 0.37)	0.04	0.06
Pandemic	White Matter	0.87 (0.42, 1.31)	0.00	0.00
Pandemic	Left Hippocampus	-0.05 (-0.51, 0.42)	0.83	0.88
Pandemic	Right Hippocampus	-0.14 (-0.64, 0.37)	0.58	0.87
Pandemic	Left Amygdala	-0.35 (-1.06, 0.36)	0.33	0.82
Pandemic	Right Amygdala	0.05 (-0.59, 0.69)	0.88	0.88
Pandemic	Cerebellum	0.12 (-0.17, 0.41)	0.41	0.82

© 2024 Weiner S et al. *JAMA Network Open*.
