# Supplementary material for: Prenatal Maternal Psychological Distress During the COVID-19 Pandemic and Newborn Brain Development
Source: JAMA Netw Open. 2024 Jun 20;7(6):e2417924. doi: 10.1001/jamanetworkopen.2024.17924 (PMC11190810; doi:10.1001/jamanetworkopen.2024.17924)
Supplement: Supplement 2. — Data Sharing Statement [file jamanetwopen-e2417924-s002.pdf]

Data Sharing Statement

Weiner. Prenatal Maternal Psychological Distress During the COVID-19 Pandemic and Newborn Brain Development. *JAMA Netw Open*. Published June 20, 2024.
doi:10.1001/jamanetworkopen.2024.17924

Data
Data available: Yes
Data types: Deidentified participant data, Data dictionary
How to access data: Data can be made available from the corresponding author upon request.
When available: With publication

Supporting Documents
Document types: None

Additional Information
Who can access the data: Data can be made available from the corresponding author upon request.
Types of analyses: Data can be made available from the corresponding author upon request.
Mechanisms of data availability: Data can be made available from the corresponding author upon request.
